# Linking the Electrical Conductivity and Non-Stoichiometry of Thin Film Ce_1−x_Zr_x_O_2−δ_ by a Resonant Nanobalance Approach

**DOI:** 10.3390/ma14040748

**Published:** 2021-02-05

**Authors:** Iurii Kogut, Alexander Wollbrink, Carsten Steiner, Hendrik Wulfmeier, Fatima-Ezzahrae El Azzouzi, Ralf Moos, Holger Fritze

**Affiliations:** 1 Institute of Energy Research and Physical Technologies, Clausthal University of Technology, 38640 Goslar, Germany; alexander.wollbrink@tu-clausthal.de (A.W.); hendrik.wulfmeier@tu-clausthal.de (H.W.); fatima.ezzahrae.el.azzouzi@tu-clausthal.de (F.-E.E.A.); holger.fritze@tu-clausthal.de (H.F.); 2 Department of Functional Materials, Bayreuth Engine Research Center (BERC), University of Bayreuth, 95440 Bayreuth, Germany; functional.materials@uni-bayreuth.de (C.S. & R.M.)

**Keywords:** ceria-zirconia solid solutions, thin films, electrical conductivity, non-stoichiometry, resonant nanobalance, thermogravimetry, reducing atmosphere, redox reactions, defect interactions, three-way catalytic converters (TWC)

## Abstract

Bulk ceria-zirconia solid solutions (Ce_1−x_Zr_x_O_2−δ_, CZO) are highly suited for application as oxygen storage materials in automotive three-way catalytic converters (TWC) due to the high levels of achievable oxygen non-stoichiometry δ. In thin film CZO, the oxygen storage properties are expected to be further enhanced. The present study addresses this aspect. CZO thin films with 0 ≤ x ≤ 1 were investigated. A unique nano-thermogravimetric method for thin films that is based on the resonant nanobalance approach for high-temperature characterization of oxygen non-stoichiometry in CZO was implemented. The high-temperature electrical conductivity and the non-stoichiometry δ of CZO were measured under oxygen partial pressures *p*O_2_ in the range of 10^−24^–0.2 bar. Markedly enhanced reducibility and electronic conductivity of CeO_2_-ZrO_2_ as compared to CeO_2−δ_ and ZrO_2_ were observed. A comparison of temperature- and *p*O_2_-dependences of the non-stoichiometry of thin films with literature data for bulk Ce_1−x_Zr_x_O_2−δ_ shows enhanced reducibility in the former. The maximum conductivity was found for Ce_0.8_Zr_0.2_O_2−δ_, whereas Ce_0.5_Zr_0.5_O_2-δ_ showed the highest non-stoichiometry, yielding δ = 0.16 at 900 °C and *p*O_2_ of 10^−14^ bar. The defect interactions in Ce_1−x_Zr_x_O_2−δ_ are analyzed in the framework of defect models for ceria and zirconia.

## 1. Introduction

Cerium oxide (ceria, CeO_2−*δ*_) and its derivatives are of great technological interest for a variety of applications, of which the catalytic control of automobile emissions prevails [1,2,3,4]. In particular, the operation of three-way catalytic converters (TWC), which was used for the exhaust gas aftertreatment of most gasoline-powered vehicles, relies on the capability of ceria-based catalyst to store and release oxygen. This capability stems from easily achievable (and reversible) non-stoichiometry *δ*, which, in CeO_2−*δ*_ at high temperatures and low oxygen partial pressures (*p*O_2_), can attain very high levels [5,6] up to the theoretical value of 0.5 (the Ce_2_O_3_ limit) [7], while maintaining the cubic crystal structure of ceria (fluorite-type). However, the theoretical maximum non-stoichiometry of CeO_2_ has never been reached experimentally so far [5,8,9,10,11,12]. The achieving of *δ* ≈ 0.01 in CeO_2−*δ*_ required strongly reducing atmospheres with *p*O_2_ < 10^−22^ bar (700 °C) or *p*O_2_ < 10^−16^ bar (900 °C) [9,11,12], and saturated values of *δ* ≈ 0.2 (900 °C; *p*O_2_ ≈ 10^−20^ bar) increasing to approximately 0.3 (1100 °C; *p*O_2_ < 10^−17^ bar) were reported in [11,12].

The release or uptake of oxygen is equivalent to the formation or recombination of oxygen vacancies, whose the concentration is directly proportional to the degree of reduction. Because oxygen vacancies are positively charged species at high temperature, the principle of electroneutrality requires the formation of opposite charge in the system—in this case, free electrons. The latter are delivered by the reduction of cerium cations from the Ce^4+^ oxidation state to Ce^3+^. Hence, the non-stoichiometric ceria is considered to be a mixed conductor with ionic contribution being realized by vacancy transport mechanism and electronic part contributed by small polaron hopping localized at neighboring Ce^4+^ and reduced Ce^3+^ cations [5,8,13]. From an application point of view, in particular, in TWCs, good oxygen storage behavior requires that the electronic conductivity is relatively high, even at high and medium oxygen partial pressures. However, pure ceria is poorly reducible in moderate reducing conditions, so that minor stoichiometric deficiencies, such as δ = 10^−4^–10^−5^, are observed at high *p*O_2_, and considerable deviations of oxygen stoichiometry only occur at very low oxygen partial pressures [5,8,9,10,11,12]. Furthermore, the long-term stability of catalytic properties of CeO_2_ at high temperatures is limited by the effect of thermal sintering and the deactivation of redox couple (aging) which reduce the oxygen storage capacity [3,14,15,16,17]. Upon the developing of ceria-based materials with tailored properties for use as the oxygen storage component in state-of-the-art TWCs, these drawbacks can be mitigated by extrinsic doping with other aliovalent (alkali, rare-earths, transition metals) or isovalent (Zr, Hf, Ti) metal cations to form solid solutions with functional point defects [14,15,16,17,18,19,20,21,22,23,24,25,26]. In this regard, the Zr-substituted ceria, i.e., Ce_1−x_Zr_x_O_2−δ_ (henceforth, CZO), has attracted the increasing interest of automobile industry and researchers as potentially the most prospective catalyst material for TWCs [17,23,24,27,28,29]. In principle, pure ceria already features a high oxygen diffusion coefficient (which implies high conductivity), a high mechanical strength, and an outstanding resistance to corrosive gases [14,17], but, in Zr-substituted ceria, these properties are further improved. By the substitution of Zr for Ce, the reduction temperature can be downshifted by 200–300 °C below that of pure CeO_2_, whereas the thermal stability against aging is extended to temperatures as high as 1000 °C that are typical for operating combustion engines [24,30]. Because of the strain induced in the crystal lattice by the substitution of smaller Zr^4+^ for larger Ce^4+^, the reduction enthalpies in Ce_1−x_Zr_x_O_2−δ_ (CZO) are substantially decreased, so that higher degrees of non-stoichiometry and superior oxygen storage capacity can be attained at higher *p*O_2_ conditions as compared to pure CeO_2_ [9,14,15,16,17,18,23,24,27,28,29,30,31,32,33,34,35]. This is because the strain favors the formation of oxygen vacancies associated with structural relaxation through a reduction of Ce^4+^ ion to a larger Ce^3+^ [9]. Hence, the addition of ZrO_2_ essentially transforms the CZO into strongly electronic conductors [9,36,37]. By the virtue of downscaling the particle sizes to submicron dimensions or confining the catalyst material in thin films, leading to extension of active surface, the catalytic properties of ceria derivatives can be further enhanced [17,24,30,38,39,40]. Because the redox reactions in ceria proceed in two serial processes, namely bulk diffusion of oxygen species and surface reactions [41], and, due to high oxygen diffusion coefficient of ceria, the catalytic processes in low-dimensional thin films with high surface-to-volume ratios are very fast, providing that surface reactions are not rate limiting. Hence, significantly faster reduction and equilibration of oxygen non-stoichiometry with the environment can be expected in comparison to conventional bulk material, especially when operated above several hundred degrees °C. By the addition of ZrO_2_, the thermal aging effect can be minimized and the extended surface morphology of thin film CZO can be preserved with no decrement in reducibility at higher temperatures [3,17,24]. These features were successfully used for the creation of fast conductometric oxygen sensors based on nanocrystalline and thin-film ceria or ceria-zirconia to be applied in exhausts or flue gases [14,15,16]. The addition of ZrO_2_ also reduces the detrimental for TWCs chemical expansion inherent for CeO_2_ [42,43]. To this end, such improved catalytic, thermo-mechanical, and chemo-physical properties in less material open prospects for material saving, device miniaturization, and cost-efficiency of modern TWC technology.

Obviously, the knowledge of mechanisms of electrical conductivity and oxygen storage in ceria-based mixed oxides at high deviations from stoichiometry are highly important for a proper operation of state-of-the-art TWCs. Extensive modelling and comprehensive reviews of electrical, dielectric, and oxygen storage properties, and defect interactions in ceria-based oxides (an introduction to defect chemistry in CeO_2_ derivatives is provided in Section 2.1) have been published in recent years [17,18,23,24,25,37,40,44,45,46,47]. It was even possible to determine *operando* by radio frequency methods the actual degree of oxygen loading [48,49], which is the key property in catalyst applications. It depends on the non-stoichiometry and it is an indirect measure of the Ce^4+^/(Ce^3+^ + Ce^4+^) ratio. It can also be derived by equilibrium data [50]. A clear correlation between the oxygen partial pressure-dependent dielectric material properties (conductivity, permittivity) and the oxygen non-stoichiometry is required in order to derive the oxygen non-stoichiometry by radio frequency measurements.

The experimental validation of the proposed models requires an appropriate correlation of oxygen non-stoichiometry and electrical conductivity of ceria-based mixed oxides in reducing and oxidizing conditions. Because the reduction is generally favored at elevated temperatures and low gas-phase oxygen potentials [18], it is important that the investigation is based on thermogravimetry. It allows for direct access to non-stoichiometry and it is particularly reliable. For thin films, a strong dependence of non-stoichiometry on oxygen partial pressure of the form *δ* ~ (*p*O_2_)^−1/2^ was reported in [51]. This was attributed to the formation of neutral oxygen vacancies or Langmuir surface adsorption on the nanoparticles. A greatly reduced reduction enthalpy in comparison to bulk samples was reported for nanostructured CeO_2−δ_ [52,53], and a higher thermal stability of mesoporous mixed CeO_2_-ZrO_2_ crystals was shown in [54]. An enhanced ionic conductivity of nanostructured Ce_0.75_Zr_0.25_O_2−δ_ was reported in [55], and a decrease of the apparent activation energy of conductivity was observed at high temperatures for nanostructured CeO_2−δ_ films [56]. These facts further justify the need to investigate the non-stoichiometry of thin ceria-based layers with high surface-to-volume ratios by means of thin film thermogravimetry, since, on the one hand, significantly lower time constants to establish equilibria should occur compared to volume samples, provided that surface processes are not limiting. On the other hand, it can be assumed that the thermal stability of thin films is higher than that of nanocrystalline materials.

Given the prominent prospective of Ce_1−x_Zr_x_O_2−δ_ for use in automotive three-way catalysts with a high oxygen storage capacity and accounting for the above-mentioned considerations, this study presents an investigation of thin film CeO_2_-ZrO_2_ mixed oxides in a broad range of compositions, from 0 to 100 mol.% of zirconia fraction. The nanobalance approach for the direct thermogravimetric investigation of the non-stoichiometry of CZO thin films was utilized. The method allows for high-precision measurements with mass resolutions as low as few nanogramms [22], which are inaccessible by means of conventional thermogravimeters that are used to study bulk materials. Both the thermogravimetric characterization and measurements of electrical conductivity of CZO thin films were performed at temperatures from 600 to 900 °C in reducing and oxidizing conditions with precise control of oxygen partial pressures (*p*O_2_) in the range of 10^−24^–0.2 bar. To the best of our knowledge, no studies of thin film CeO_2_-ZrO_2_ with that broad range of compositions have been reported to date.

## 2. Theoretical Background, Samples Preparation and Experimental Methods

### 2.1. Defect Interactions in CeO_2−δ_ and Ce_1−x_Zr_x_O_2−δ_

In this section, a short introduction to defect chemistry of ceria-based compounds is provided. Although the discussed defect interactions are specific to cubic fluorite-type structure, the defect model of pure CeO_2_ fairly applies also to cubic and tetragonal CZO and monoclinic ZrO_2_ [32,57]. Subsequently, only the expressions for dependences of electrical conductivity (*σ*), oxygen non-stoichiometry (*δ*), and defect concentrations (in square brackets) on oxygen partial pressure (*p*O_2_) and temperature (*T*) are provided without derivation in order to highlight the most relevant relations for a discussion of the experimental results.

In the fluorite-type cubic structure, the formation of intrinsic anion-Frenkel defects is favored, i.e., equal amounts of doubly charged oxygen vacancies and interstitial oxygen ions are formed:

(1) $$O_{O}^{\text{×}}↔{\;V}_{O}^{\;\cdot \cdot }{\;+\;O}_{i}^{\prime \prime },$$

where, written in Kröger–Vink notation, $O_{O}^{\text{×}}$, $V_{O}^{\;\cdot \cdot }$ and $O_{i}^{\prime \prime }$ denote the neutral oxygen atoms, the doubly positively charged oxygen vacancies and the doubly negatively charged interstitial oxygen ions, respectively. Thereby, the charge is given with respect to the lattice. Because $O_{i}^{\prime \prime }$ are less mobile than $V_{O}^{\;\cdot \cdot }$, the motion of the latter dominates the electrical conductivity, and pure stoichiometric CeO_2_ is intrinsically an ionic conductor [32].

At elevated temperatures in reducing atmosphere, the stoichiometry of ceria deviates due to the release of oxygen. Thereby, doubly positively charged oxygen vacancies and compensating electrons are formed [12]:

(2) $${2\mathrm{Ce}}_{\mathrm{Ce}}^{\text{×}}{\;+\;O}_{O}^{\text{×}}↔{\;V}_{O}^{\;\cdot \cdot }{\;+\;2\mathrm{Ce}}_{\mathrm{Ce}}^{\prime }\;+\;\frac{1}{2}O_{2}\;(g),$$

where ${\mathrm{Ce}}_{\mathrm{Ce}}^{\prime }$ (i.e., Ce^3+^) describes an electron (a small polaron [5]) that is localized in the vicinity of a Ce ion. In this case, the concentration of electrons is related to the non-stoichiometry by [${\mathrm{Ce}}_{\mathrm{Ce}}^{\prime }$] = 2[$V_{O}^{\;\cdot \cdot }$] = 2δ. In the broad range of *p*O_2_, the conductivity of non-stoichiometric ceria becomes mixed with the ionic part from anion motion by vacancy mechanism and electronic contribution from small polaron hopping between cerium ions [5,8,12]. At high *p*O_2_, the non-stoichiometry is small, the ${\mathrm{Ce}}_{\mathrm{Ce}}^{\text{×}}$ (neutral cerium with respect to the lattice, Ce^4+^) and $O_{O}^{\text{×}}$ sites are nearly fully occupied, the amounts of extrinsically generated electrons and vacancies are small, and the formed defects do not interact. Thus, the defect chemistry is well-described in the framework of an isolated defect model. In this reduction regime, the concentrations [${\mathrm{Ce}}_{\mathrm{Ce}}^{\prime }$] and [$V_{O}^{\;\cdot \cdot }$], and, accordingly, the electrical conductivity *σ* vary as (*p*O_2_)^−1/6^ [18], but the conductivity is dominantly n-type electronic, due to the much higher mobility of electrons when compared to oxygen vacancies. At lower *p*O_2_ and larger non-stoichiometry, the concentration of available electrons due to Ce^4+^/Ce^3+^ reduction rises, and defect interactions can no longer be neglected. Early literature [5,8] suggested that, in this regime, the probability of recombination of excess electrons with $V_{O}^{\cdot \cdot }$ increases and singly ionized oxygen vacancies (with concentrations [$V_{O}^{\;\cdot }$] = [${\mathrm{Ce}}_{\mathrm{Ce}}^{\prime }$]) are formed:

(3) $${\mathrm{Ce}}_{\mathrm{Ce}}^{\text{×}}{\;+\;O}_{O}^{\text{×}}↔{\;V}_{O}^{\;\cdot }{\;+\;\mathrm{Ce}}_{\mathrm{Ce}}^{\prime }\;+\;\frac{1}{2}O_{2}\left(g\right)\;\mathrm{or}\;V_{O}^{\;\cdot }↔{\;V}_{O}^{\;\cdot \cdot }{\;+\;e}^{\prime }$$

In this case, the concentrations and conductivity obey a (*p*O_2_)^−1/4^-dependence. However, more recent studies suggested the formation of dimer ${{(\mathrm{Ce}}_{\mathrm{Ce}}^{\prime }V_{O}^{\;\cdot \cdot })}^{\cdot }\;$defect associations, qualitatively equivalent to $V_{O}^{\;\cdot }$, rather than recombination of Ce^3+^ with oxygen vacancies in this non-stoichiometry domain [18,57]:

(4) $${2\mathrm{Ce}}_{\mathrm{Ce}}^{\text{×}}{\;+\;O}_{O}^{\text{×}}↔{\;\mathrm{Ce}}_{\mathrm{Ce}}^{\prime }{\;+\;(\mathrm{Ce}}_{\mathrm{Ce}}^{\prime }V_{O}^{\;\cdot \cdot }{)}^{\cdot }+\;\frac{1}{2}O_{2}\left(g\right)$$

Furthermore, the formation of neutral trimer defect associations is possible in reduced CeO_2_ for larger non-stoichiometries, e.g., δ > 0.01:

(5) $${2\mathrm{Ce}}_{\mathrm{Ce}}^{\prime }{\;+\;V}_{O}^{\;\cdot \cdot }↔{\left({\mathrm{Ce}}_{\mathrm{Ce}}^{\prime }{\text{–}V}_{O}^{\;\cdot \cdot }{\text{–}\mathrm{Ce}}_{\mathrm{Ce}}^{\prime }\right)}^{\text{×}}$$

Their concentration varies with (*p*O_2_)^−1/2^, and the total background of oxygen vacancies is then composed of isolated $V_{O}^{\;\cdot \cdot }$ and trimers, which, at intermediate oxygen activities, give rise to steeper *p*O_2_-dependence of non-stoichiometry *δ* with slopes between −1/6 and −1/2 [18,58]. Finally, at large deviations from stoichiometry (i.e., at even lower *p*O_2_), the hopping of small polarons between ${\mathrm{Ce}}_{\mathrm{Ce}}^{\prime }$ and ${\mathrm{Ce}}_{\mathrm{Ce}}^{\text{×}}$ is hindered, because the probability of hops from ${\mathrm{Ce}}_{\mathrm{Ce}}^{\prime }$ sites to ${\mathrm{Ce}}_{\mathrm{Ce}}^{\text{×}}$ sites is reduced due to an insufficient amount of available ${\mathrm{Ce}}_{\mathrm{Ce}}^{\text{×}}$, when the concentrations become [${\mathrm{Ce}}_{\mathrm{Ce}}^{\prime }$] ≥ [${\mathrm{Ce}}_{\mathrm{Ce}}^{\text{×}}$]. Hence, the polaronic conductivity of CeO_2−δ_ should transit through a maximum [20,32].

The defect interactions in CeO_2−δ_ depend on the purity of the material [12,18,32,35,55,57]. Impurity cations with lower valence with respect to Ce^4+^ and similar in size (acceptors A, e.g., Ca^2+^, Y^3+^) substitute on Ce sites (in Kröger–Vink notation denoted as $A_{\mathrm{Ce}}^{\prime \prime }$ or $A_{\mathrm{Ce}}^{\prime }$, respectively) favoring the formation of extrinsic oxygen vacancies:

(6) $$\mathrm{AO}\;\overset{{\mathrm{CeO}}_{2}}{↔}{\;A}_{\mathrm{Ce}}^{\prime \prime }{\;+\;V}_{O}^{\;\cdot \cdot }{\;+\;O}_{O}^{\text{×}}$$

(7) $$A_{2}O_{3}\overset{{\mathrm{CeO}}_{2}}{↔}{\;2A}_{\mathrm{Ce}}^{\prime }{\;+\;V}_{O}^{\;\cdot \cdot }{\;+\;3O}_{O}^{\text{×}}$$

The concentration of extrinsic vacancies is fixed by that of dopants (2[$V_{O}^{\;\cdot \cdot }$] = [$A_{\mathrm{Ce}}^{\prime }$]). At relatively low temperatures and low non-stoichiometry, it is considered to be constant, but is substantially larger than that of intrinsic vacancies or electrons generated by the reduction of cerium. In this case, the conductivity follows the (*p*O_2_)^−1/4^ dependence [18,59,60]. Impurities with higher valence (donors D, e.g., Nb^5+^) lead to the suppression of $V_{O}^{\;\cdot \cdot }$ and formation of interstitial negatively charged oxygen ions $O_{i}^{\prime \prime }$. They become dominant defects in oxidizing conditions ([$D_{\mathrm{Ce}}^{\cdot }$] = 2[$O_{i}^{\prime \prime }$]). Electronic compensation ([$D_{\mathrm{Ce}}^{\cdot }$] = [${\mathrm{Ce}}_{\mathrm{Ce}}^{\prime }$]) is observed in reducing atmospheres [60].

As mentioned earlier, the defects in the CeO_2_-ZrO_2_ system are described in a similar way as for ceria. However, in contrast to Ce, Zr always retains the 4+ charge state and, therefore, cannot form the charge compensating defects. The formation of $V_{O}^{\;\cdot \cdot }$ according to:

(8) $${\mathrm{CeO}}_{2}\;\;\;\overset{{\mathrm{ZrO}}_{2}}{↔}{\;\mathrm{Ce}}_{\mathrm{Zr}}^{\text{×}}{\;+\;2V}_{O}^{\;\cdot \cdot }{\;+\;2O}_{i}^{\prime \prime }$$

is attributed to strain that developed upon Ce substitution for Zr due to substantial difference in the ionic radii of the two (1.01 Å vs. 0.80 Å, respectively [35]). The crystal lattice gets strained, as smaller Zr ion prefers the seven-fold coordination in contrast to the 8-fold coordination of the fluorite cation (Ce^4+^). The developed strain favors the formation of oxygen vacancies associated with structural relaxation through reduction of Ce^4+^ ion to a larger Ce^3+^ [9], i.e., Zr cations strongly interact with oxygen vacancies, leading to a more negative defect interaction energy in zirconia-doped ceria, thus, facilitating the reduction of Ce^4+^ to Ce^3+^ [9,36,37]. Like in pure ceria, in Ce_1−x_Zr_x_O_2−δ_ the concentrations [${\mathrm{Ce}}_{\mathrm{Ce}}^{\prime }$] and [$V_{O}^{\;\cdot \cdot }$], and the conductivity, vary as (*p*O_2_)^−1/6^, and, at very low *p*O_2_, the dependence with −1/4 slope, followed by a maximum, should be expected [32].

It is worth noting that different *p*O_2_-dependences of charge carrier concentrations and conductivities in ceria-based oxides were derived when accounting for different dominant defect interactions in ceria, zirconia, and CZO, yielding slopes of ±1/2, ±1/3, ±1/4, ±1/5, and ±1/6 for p-type and n-type conductivities [32,57,61,62,63]. Obviously, one would rarely observe the ideal slopes in practice. The dominant defects always occur at the background of minor defect configurations, and the electrical conductivity should, hence, be regarded as a sum of contributions from all of the charged species. Concerning the thermogravimetric assessment of non-stoichiometry, the analysis is complicated by the very weak deviations of *δ* at high *p*O_2_, which lead to *n* > −6 in δ ∝ (*p*O_2_)^1/*n*^ dependences [18].

The microstructural, bulk, and surficial properties of the studied material also play an important role. For example, in thin films, the redox kinetics are typically limited by the surface reaction rate, which may lead to *p*O_2_-dependent conductivities with a slope of −0.35 [41]. In [56], the nanostructured CeO_2-δ_ thin film and bulk specimens showed a *p*O_2_-independent conductivity (ionic contribution) turning to the dependences with −1/4 slope at lowered *p*O_2_, characteristic for extrinsic conductivity. As the temperature increased above 800 °C, the purely intrinsic conductivity with (*p*O_2_)^−1/6^ dependence was observed. In [55], the unexpectedly high ionic contribution at high *p*O_2_ was attributed to a higher degree of disorder and lower atomic density at the interfaces of nanocrystallites in Ce_0.75_Zr_0.25_O_2−δ_. A (*p*O_2_)^−1/5^-dependence of the total conductivity was observed in that study.

The present paper is aimed at the experimental correlation of *p*O_2_-dependences of electrical conductivity and oxygen non-stoichiometry of thin-film Ce_1−x_Zr_x_O_2−δ_ and the validation of the applicability of defect models for bulk CZO. Due to the rather bulk-like thickness of the films studied here, the question needs to be answered as to whether the effects due to low dimensions come into play. Thereby, the study is expected to provide hints that are relevant to an extension of defect model, which are specifically adapted for Ce_1-x_Zr_x_O_2-δ_ thin films with high surface-to-volume ratios, when required.

### 2.2. Sample Preparation

The starting Ce_1−x_Zr_x_O_2_ solid solutions for the study were prepared by solid-state reaction using the following routine. First, the CeO_2_ (99.99% purity, Chempur GmbH, Karlsruhe, Germany) and ZrO_2_—2% HfO_2_ powders (99.95% purity, same supplier) were dried at 120 °C for 24 h to remove the possible moisture contained in powders, and weighed in the stoichiometric ratios that correspond to *x* = 0, 0.2, 0.33, 0.5, 0.67, and 1. Hafnia, which is present in relatively high concentrations, should not influence the properties of CZO, because Hf is isovalent with Zr (4+) and the eight-fold coordination ionic radii of both are similar (0.83 and 0.84, respectively) [64]; however, the hafnia content was considered when calculating the stoichiometry. The powders were then mixed and homogenized by milling in a planetary ball mill (Fritsch Pulverisette 5) at 400 rpm while using a 3.5%—MgO hardened ZrO_2_ crucible and 5%—Y_2_O_3_ stabilized zirconia balls. The homogenization was performed in two runs, each 2 min. long, with a 15 min. pause in between. Following that, the CeO_2_-ZrO_2_ powder mixtures were loaded in Al_2_O_3_ annealing beakers, heated at the rate of 5 K/min. (Nabertherm LHT08/17 chamber furnace, Nabertherm GmbH, Lilienthal, Germany), calcined for 12 h at 1650 °C, and then cooled with the furnace time constant. The calcined ingots were ball-milled again in nine 5 min. milling—20 min. pause cycles using the above-mentioned parameters, and the resulting powders were heat-treated for 5 h at 623 °C with 5 K/min. heating and cooling rates. Subsequently, the powders were cold-pressed into bulk Ø13 mm (2 g) and Ø20 mm (4 g) pellets under the uniaxial force of 20 kN (Raptor WPS 10C), applied for 10 min. and followed by the 5 min. long pressure release. These pellets were sintered at 1650 °C for 12 h while using the same Al_2_O_3_ beakers and process parameters as for the calcination stage. Finally, the sintered material was subjected to heat treatment at 623 °C for 24 h with 5 K/min. heating and cooling steps. For sake of clarity, the studied ceria-zirconia solid solutions will be henceforth named CZO-0, CZO-20, CZO-33, CZO-50, CZO-67, and CZO-100 with numbers standing for the zirconia content in mol.%, as provided in Table 1.

The sintered bulk Ce_1−x_Zr_x_O_2_ materials were characterized by X-ray diffraction (XRD, Bruker D8 Advance (Bruker AXS GmbH, Karlsruhe, Germany) with Cu anode and germanium Kα_1_ monochromator (λ = 1.5406 Å), and 1D Lynxeye detector) in Bragg–Brentano geometry within 2θ = 25°–75° with 0.02° resolution to justify the formation of desired phases with respect to zirconia content. The crystallinity of the thin films was characterized after all the experiments using a Siemens D5005 X-ray diffractometer (Cu K_α1_) (Siemens AG, Karlsruhe, Germany) that was equipped with a scintillation detector, and a Ni shield placed in the beam path in front of the detector to suppress Cu-K_β_ reflections.

Laser-assisted inductively coupled plasma mass spectrometry (ICP-MS, Element XR, ThermoScientific, Bremen, Germany) was used in order to determine the impurity content in the sintered targets of edge compositions CeO_2_ and ZrO_2_ (see Section 3.1).

**Figure 1:**
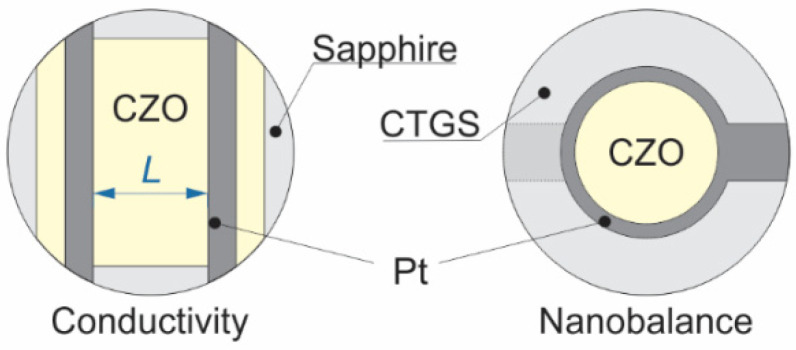
Scheme of Ce_1−x_Zr_x_O_2_ thin film samples deposited on sapphire and Ca_3_TaGa_3_Si_2_O_14_ (CTGS) substrates used for studies of electrical conductivity and non-stoichiometry respectively.

The Ce_1−x_Zr_x_O_2_ thin films were fabricated by pulsed laser deposition (PLD) ablating the sintered CZO targets onto selected substrates (Figure 1) using the 248 nm excimer KrF laser (COMPex, Coherent, Inc., Santa Clara, CA, USA) with a pulse energy of 200 mJ and repetition rate of 10 Hz. The deposition was performed without additional heating of the substrate, i.e., at or near room temperature, in vacuum with chamber pressures of 5 × 10^−6^ –10^−5^ mbar during ablation. The coloration of as-deposited films differed from that of the sputter targets, changing from dark-gray-bluish (*x* = 0; 0.2) or greenish-dark-brown (0.33 ≤ *x* ≤ 0.67) to black for pure ZrO_2_, indicating oxygen being deficit in them [65]. In order to recover the oxygen stoichiometry, the CZO films were annealed in air at the temperature of 900 °C for 1 h (heating rate of 2 K/min.). This resulted in a pale yellowish tint, which was similar to that of the starting material. The actual chemical composition of annealed thin films, normalized to cerium content, nearly perfectly matched the nominal one, as determined by the energy-dispersive X-ray spectroscopy (EDS, CamScan 44, CamScan Electron Optics Ltd., Waterbeach, UK) (Table 1).

### 2.3. Experimental Methods

For electrical conductivity measurements, the 8 × 8 mm^2^ CZO films with thicknesses *t* (Table 1) were sputtered onto a 0.8 mm thick and Ø10 mm single crystal sapphire substrates, purchased from SITUS Technicals GmbH. The conductivity measurements were performed in the lateral two-contact configuration, in which two electrode stripes were applied over the CZO film surface (Figure 1) at the distances *L* (Table 1). The electrodes were deposited after the above-mentioned annealing by the mesh printing of Pt paste followed by firing at 900 °C for 1 h with a heating rate of 2 K/min. The choice of single crystalline sapphire substrates for electrical conductivity measurements is based on sapphire’s extremely low electrical conductivity and excellent long-time stability with respect to reducing atmosphere and high temperatures [66,67]. Although in lateral measurements configuration the electrical conductivity of the substrate might impact the overall resistance of film and substrate and, thereby, falsify the property of interest, i.e., the conductivity of the film. In the case of the lowest expected film conductivity, i.e., for ZrO_2_, the overall resistance of film and substrate at 900 °C is found to be approximately 125 MΩ. Taking conductivity data for sapphire at 900 °C from [67] and the dimensions of the substrate, a much higher resistance of about 250 GΩ is expected. Hence, the influence of sapphire substrates on the measured resistance of CZO thin films is negligible.

For the determination of non-stoichiometry, a resonant nanobalance approach to thermogravimetric characterization of Ce_1−x_Zr_x_O_2−δ_ thin films was applied. The plates of piezoelectric *y*-cut single crystals CTGS (Ca_3_TaGa_3_Si_2_O_14_, Fomos, Russia; 0.25–0.3 mm thick and Ø10 mm disks) were used as resonant nanobalances [68,69]. The resonators were operated in the fundamental tone of thickness-shear mode with “unloaded” resonance frequency *f*_R_ of ca. 5 MHz. The ca. 3 µm thick keyhole-shaped Ø6 mm electrodes were applied on both faces of the CTGS disks by mesh printing of the Pt paste. After that, the Ø5 mm CZO thin films, the “loaded mass”, were deposited by PLD onto the Pt electrodes (Figure 1) in several runs until the mass load on both sides of CTGS resonators became nearly equal.

The electrical conductivity and non-stoichiometry measurements were both performed in isothermal conditions at 600, 700, 800, and 900 °C, under *p*O_2_ in the range of 10^−24^–0.2 bar. A special sample holder was designed to characterize all the thin film CZO samples at once, thereby excluding differences in the experimental conditions that were potentially caused by different runs. Further, possible temperature gradients over the measured samples are minimized. The precise temperature control is enabled via the Type S thermocouple, located in the vicinity of the measured samples, and a Pt100 thermoresistor was used to compensate the thermocouple’s cold end temperature fluctuations. The oxygen partial pressure in the *p*O_2_ range of 10^−24^–10^−4^ bar was adjusted by an oxygen ion pump [70], adding small amounts of oxygen to the reducing gas mixture of 99.5% Ar—0.5% H_2_ flowing through the furnace at a rate of 20 mL/min. Oxygen partial pressures between 0.01 and 0.2 bar were adjusted by supplying the appropriate Ar-O_2_ mixture in the furnace. Note that the oxygen ion pump and the oxygen sensor are operated at 700 °C and 600–700 °C, respectively, and appropriate corrections of oxygen partial pressures are required for the results that were obtained at 800 °C and 900 °C isotherms [71].

**Figure 2:**
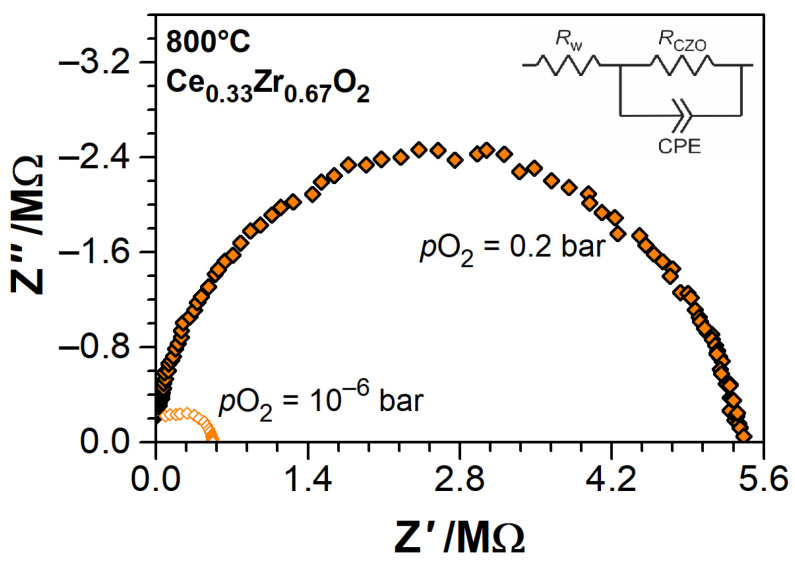
Typical impedance spectra of Ce_1−x_Zr_x_O_2_ thin films in lateral measurement configuration and a corresponding equivalent circuit with *R*_w_ being the negligible resistance of lead wires and *R*_CZO_ standing for the resistance of the Ce_1−x_Zr_x_O_2−δ_ (CZO) thin films. Z’ and Z’’ are the real and imaginary parts of impedance, respectively.

The electrical conductivity of Ce_1−x_Zr_x_O_2−δ_ thin films was evaluated by means of electrical impedance spectroscopy (EIS) while using the SI 1260 impedance spectrometer, assisted by the high-impedance measuring bridge SI 1296 (Solartron Analytical, Leicester, UK). The impedance spectra were acquired in the frequency range of 1–10^6^ Hz with an excitation AC voltage of 50 mV. Inferring from the obtained single semicircle EIS features (Figure 2), the electrical properties of CZO samples were modeled by a single equivalent circuit of bulk resistance *R*_CZO_ in parallel to constant phase element (CPE). The bulk resistance of CZO was derived by the least squares fitting of model parameters to EIS, and the electrical conductivity *σ*_B_ (*T*, *p*O_2_) was calculated while using the CZO thin films dimensions.

Additionally, the DC resistances of Ce_1−x_Zr_x_O_2−δ_ thin films were measured (Keithley DVM 2700 digital multimeter, Keithley Instruments, Solon, OH, USA) for comparison. Only minor discrepancies between the EIS and DC derived resistances were observed (see Figure 5 in Section 3.3). Furthermore, one DC resistance measurement lasts some fractions of a second, whereas the acquisition of EIS spectra requires a few minutes. The oxygen partial pressures were recorded each 3 s, and the temperature in the chamber was measured before and after each conductivity measurement (either EIS or DC). Hence, the instantaneous conditions upon DC measurements are considered to be stable and the DC results will mostly be, therefore, referred in the following.

The electrical impedance of the “loaded” CTGS resonators was measured by resonant piezoelectric spectroscopy in the vicinity of their resonance frequency [68,72] using a high-speed network analyzer E5100A in order to determine the mass changes of the deposited CZO layers (Agilent, Santa Clara, CA, USA). The resonance frequency is regarded as the frequency at the maximum of the admittance peak and was determined by fitting a Lorentz function to the conductance in the vicinity of the resonant peak. Frequency fluctuations of the CZO-coated resonators, not related to the mass changes, including the temperature-induced ones, were compensated by subtracting the simultaneously acquired frequency changes of the reference “unloaded” CTGS resonator.

It is important that the thermal stability of CTGS is confirmed in the context of the high-temperature nano-gravimetric experiments performed here. Such resonators exhibit nearly unchanged resonance behavior at least after more than one year of uninterrupted operation at 1000 °C in air. In particular, the resonance frequency shows a relative shift of only 0.4 % during this period [73]. Moreover, the defect chemistry and options for miniaturization of these resonators are known [69,74,75].

In the resonant nanobalance approach, the shift of the resonance frequency (Δ*f*_R_) of a resonator is directly proportional to the change in mass load Δ*m* and it can be approximated by Sauerbrey’s relation [69,76]. Several challenges that are due to the operation of the resonators at high temperatures have to be considered. First, the mass sensitivity depends on temperature and it is, e.g., affected by the effective resonator area, which is required to calculate the effective mass sensitivity. The aforementioned area is, in principle, determined by the quality factor of the resonator, the electrode diameter and thickness, the curvature of the resonator, etc. Its calculation that is based on materials parameters is error-prone. Therefore, a one-dimensional physical model, as described in [68], is used to model the impedance spectra that are fitted to the related experimental data. The fit procedure results in reasonable values of the physical properties, such as elastic constants and effective viscosity, as published in [73], thereby confirming the validity of the fit approach. Another point is that the change in mass results from a change in density of the CZO film. The aforementioned model includes not only the CTGS resonator, but also external films whose density is taken into account. As a consequence, the fit procedure includes the dimensions and material properties of the CZO films. To finally determine the required dependence of the frequency on the film density *ρ*_CZO_, the latter is slightly varied in the model in order to determine the density sensitivity

(9) $$S_{D\;}=\;\frac{{d\rho }_{\mathrm{CZO}}}{{df}_{R}}$$

The calculation must be performed for each individual CZO coated resonator, since different values result from different resonator parameters. The values for different resonators range from about −2.0 × 10^−2^ to −3.5 × 10^−2^ kg/m^3^/Hz. Thereby, the differences between the individual resonators are much larger than the temperature dependent changes of a single resonator of approximately 1.2% for temperatures in the range from 500 to 900 °C. It should be noted that the aforementioned physical model was used in a similar way to determine the CZO film thickness. The resulting values for the frequency shift related to the film thickness increase range from about −15 to −22 pm/Hz. Because the frequency of the resonators before and after deposition was determined at room temperature, the calculation was made for this condition. The values for *d_CZO_* in Table 1 were calculated while using this relation of film thickness and frequency. Obviously, the major limitation in such an approach is related to the accuracy of temperature measurements by thermocouple and the location of particular CZO-coated resonator relative to the references. Our analysis showed that therewith related uncertainty of measured frequency was up to ±100 Hz (due to temperature drifts within about ±0.5 K), which results in approximately 3 to 7% relative error in determination of non-stoichiometry.

In Ce_1−*x*_Zr*_x_*O_2−*δ*_ that was exposed to reducing or oxidizing atmosphere, the mass changes are due to the release or uptake of oxygen, as described above. With the known density sensitivity, the oxygen non-stoichiometry that develops in CZO can be calculated via the mass ratios:

(10) $$\delta \;=\;-\frac{M_{\mathrm{CZO}}}{M_{O}}\frac{\Delta m}{m_{0}}=\;-\frac{M_{\mathrm{CZO}}}{M_{O}}S_{D}\frac{\Delta f_{R}}{\rho_{\mathrm{CZO}}},$$

where *M*_CZO_ and *M*_O_ are the molar masses of Ce_1−*x*_Zr*_x_*O_2_ and oxygen, respectively, and *ρ*_CZO_ is the theoretical density of CeO_2_-ZrO_2_ solid solutions that account for the actual fractions of ceria and zirconia.

## 3. Experimental Results

### 3.1. Characterization of Ce_1−x_Zr_x_O_2_ Specimens

**Figure 3:**
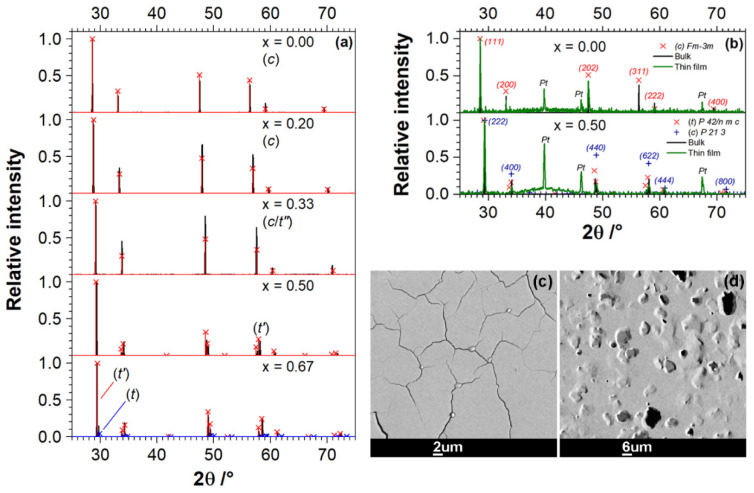
X-ray diffractograms of sintered bulk Ce_1−x_Zr_x_O_2_ targets (**a**), CZO-0, and CZO-50 thin films (**b**) and the back-scattered electron (BSE)-mode SEM images of CZO-0 (**c**) and CZO-67 (**d**) thin films deposited and annealed on single crystal sapphire substrate for electrical conductivity measurements. In (**a**,**b**), the red crosses mark the cubic *c* (*Fm-3m*) or tetragonal *t’* phases for Ce-rich and Zr-rich samples, respectively. In (**a**), the blue crosses mark the stable tetragonal *t* phase in bulk Ce_0.33_Zr_0.67_O_2_. In (**b**), the blue pluses indicate the positions for reflections of metastable primitive cubic *P 21 3* phase [82,83].

The X-ray diffraction patterns confirmed the cubic phase of CZO-0, CZO-20, CZO-33, tetragonal phase of CZO-50, CZO-67 and monoclinic phase of ZrO_2_ (not shown) in the bulk Ce_1−x_Zr_x_O_2_ PLD-targets (Figure 3a). Consistent with the literature, the sintered pellets of CZO-0 and CZO-20 were single phase polycrystalline materials with CaF_2_ fluorite type cubic symmetry [77,78], whereas the co-existence of minor metastable (*t’’*)-phase was found in CZO-33 [79]. The CZO-50 was composed of a single metastable tetragonal *t’* phase [80], and stable cubic and tetragonal phases (*c* + *t*) were found to co-exist with metastable *t’* phase [81] in CZO-67 (Figure 3a; the XRD peak at 2θ of 29.5°). We note the shift of the diffraction peaks towards larger 2θ in cubic CZO (Figure 3)), which indicates the decrease of their lattice parameter with increasing zirconia fraction, as expected from literature on Ce_1−*x*_Zr*_x_*O_2_ [9]. Figure 3b provides the comparison of XRD data of thin films (pure CeO_2_ (CZO-0) and CZO-50 are shown) with that of starting bulk targets. Similar to bulk material, the CZO-0 film crystallized in the face-centered cubic phase of the CaF_2_-type (*Fm*3*m*) [77,78,80]. However, the two relatively strong reflections (200) and (311), as observed in bulk CZO-0, are weaker or not present in the thin film sample. Presumably, this feature indicates preferential grain orientation or stress in the film that is deposited on sapphire substrate. As expected from the literature, the films of Ce_1−x_Zr_x_O_2_ mixed oxides predominantly crystallized in the tetragonal *P 42*/*n m c* phase, showing all of the reflections already found for the starting bulk materials [80]. The analysis of the XRD patterns of mixed oxide thin films indicated that the peaks are prone to one-side broadening with increasing Ce-content, which implies the coexistence of another less pronounced CZO phase in them. Based on the 2θ positions of this broadening, in particular, as observed for CZO-50, the further analysis infers that this phase crystallizes in primitive *P 21 3* cubic symmetry, as reported for metastable *κ*-CeZrO_4_ in [82,83] (Figure 3b). The formation of a cubic phase in thin films of Ce-rich ceria-zirconia mixed oxides is consistent with the observations for bulk sintered targets and literature data [77,78]. The reflections of Pt-Electrodes were also resolved by XRD [84], since the cross-section of the X-ray beam covered the area exceeding that of the films. Crystallization in monoclinic *P1 21/c 1* phase, which matches that of bulk target, was confirmed for pure ZrO_2_ thin films [85].

The ICP-MS studies of sintered CeO_2_ and ZrO_2_ targets showed the largest concentrations for SiO_2_ (450 and 660 ppm, respectively), Al_2_O_3_ (120 and 512 ppm), Fe_2_O_3_ (ca. 200 ppm in ZrO_2_), and CaO (80 and 100 ppm). The content of other impurities (La_2_O_3_, Y_2_O_3_, other rare earth cations) was negligible. In general, the CeO_2_ expectedly contained fewer impurities than ZrO_2_. The impurity concentrations of few hundred ppm are sufficient to affect the electrical conductivity of ceria and its derivatives [35]. As follows from Equations (6) and (7), the lower valence Al, Ca, and Fe cations will induce the formation of charge compensating oxygen vacancies and stronger *p*O_2_-dependences of conductivity with higher slopes. Silicon should not affect the electroneutrality of CZO, as Si is isovalent to Ce and Zr. However, while assuming that Si and Al substitute on Ce/Zr sites (though Si has a very small ionic radius and energy of doping Al into CeO_2_ is extremely high [25]), these elements with small ionic radii may substantially distort the host lattice, which favors the formation of extrinsic oxygen vacancies and leads to a strong decrease (by about 0.7 eV) of the reduction energy of ceria [25]. Besides, iron can reduce from the Fe^3+^ to Fe^2+^ state, thus contributing to the oxygen loss and total electrical conductivity of host CZO [86]. Hence, complex defect interactions and the departure of *p*O_2_-dependences of non-stoichiometry and conductivity of CZO from the theoretical ones should be anticipated.

The SEM-EDS study confirmed the anticipated chemical composition of Ce_1−x_Zr_x_O_2_ thin films that nearly perfectly matched that of the starting material (Table 1). The back-scattered electron (BSE) imaging, as, for example, for pure CeO_2_ and CZO-67 films presented in Figure 3c,d, showed no contrasts that would be indicative for secondary impurity phases. Finite cracks, but virtually no porosity, are observed in purely cubic ceria-rich thin films at room temperature (Figure 3c). However, at elevated temperature, the conductivity of CeO_2_ is essentially in agreement with literature values (see Section 3.2). This observation is a strong indicator of closed cracks at high temperatures. The indication is supported by the thermal expansion of film and substrate. Taking the values for alumina from the manufacturer [87] and for ceria from [88,89], a thermal mismatch between film and substrate of Δα = α_ceria_ − α_alumina_ ≈ 5 × 10^−6^ K^−1^ is to be estimated. For the given crack width and distance, a temperature increase of 600 K results in almost and/or partially closed cracks. In addition, the chemical expansion of ceria causes cracks to close further as *p*O_2_ decreases [90]. Therefore, it can be concluded that there is no significant disturbance of the lateral film resistance. The small spherical inclusions within the cracks of pure CeO_2_ film (Figure 3c) were determined to be cerium (III) oxide (Ce_2_O_3_). In tetragonal Zr-rich layers, the readily detectable roughness and microscale porosity developed with increasing Zr content without cracking (Figure 3d). In any case, no exfoliation of the CZO films from sapphire substrates was noticed before and after all long-time experiments in reducing atmosphere and at high temperatures.

### 3.2. Electrical Conductivity of Ce_1−x_Zr_x_O_2−δ_ Thin Films as a Function of Temperature

The temperature dependences of the electrical conductivity of Ce_1−x_Zr_x_O_2−δ_ thin films were measured at heating and cooling between the isotherms applied during the measurements of *p*O_2_-dependences of conductivity. Prior to heating/cooling, the samples were stabilized at the corresponding isotherm for at least 12 h in synthetic air. The latter condition was set by adjusting the composition of an Ar-O_2_ flow until the oxygen sensor measured log(*p*O_2_/bar) ≈ −0.7.

The impedance spectra of all Ce_1−x_Zr_x_O_2−δ_ thin films featured one semicircle on the Nyquist plots in the sweep frequency range of 1 Hz–1 MHz (Figure 2) at all temperatures and oxygen partial pressures. This suggests that either the electrical transport in CZO films is limited by the bulk grain conduction or the grain-boundary effects are superimposed with bulk conduction, as noted in [55,91] for nanostructured films. The literature predicts the domination of grain boundaries effects in the electrical conductivity of nanostructured CZO [55,91,92,93]. However, no submicron features were resolved by the SEM of our ceria-zirconia films, which are rather well compacted microscopic polycrystalline grains, but with loose integrity on the macroscopic level (Figure 3c,d). The contributions from the grain interiors and boundaries in the EIS can be separated by modeling the spectra with two R-CPE circuits in serial connection and then comparing the derived capacitances. However, in the lateral measurement configuration, the capacitance of the system cannot be determined, because the volume of material and space between the electrodes is essentially indefinite, which renders the measured capacitance into stray capacitance of the measuring device (~2 pF). Therefore, it was chosen in following, to consider the total electrical conductivity of CZO thin films and model it with a single R-CPE circuit. The CPE parameters were fixed to initially found values at *p*O_2_ = 0.2 bar, and the *R*_CZO_ was varied (the intersection of a semicircle with *Z’* axis was determined). As mentioned earlier, the DC conductivity measurements are mostly further referred.

**Figure 4:**
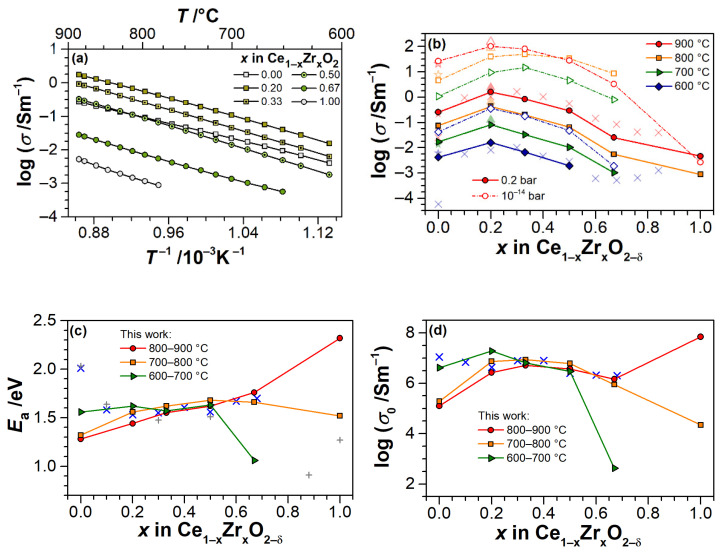
Electrical conductivity of Ce_1−x_Zr_x_O_2_ thin films as a function of temperature and Zr fraction: (**a**) obtained during cooling in air; (**b**) variation with Zr fraction in air (solid symbols) and at *p*O_2_ = 10^−14^ bar (open symbols) in comparison to CeO_2−δ_ nanofilms (Kosacki, et al. [56], stars), nanostructured bulk Ce_0.8_Zr_0.2_O_2−δ_ (Chen, et al. [32], up-triangles), and bulk Ce_1−x_Zr_x_O_2_ (Chiodelli, et al. [35], crosses, air); (**c**) dependences of *E*_a_ on *x* in comparison to data from Chiodelli, et al. [35] (crosses) and from Lee, et al. [63] (pluses) and (**d**) dependences of *σ*_0_ on *x* compared to data from Chiodelli, et al. [35] (crosses).

Figure 4a shows the temperature dependences of the electrical conductivity of Ce_1−x_Zr_x_O_2−δ_ thin films in a log(*σ*)-*T*^−1^ scale, measured at cooling from 900 to 600 °C in synthetic air. The dependences show a slight curvature with a kink at about 700–750 °C, which is especially visible for CeO_2−δ_ and CZO-67 films. Linear dependences (not shown) were observed at heating between the isotherms.

The analysis of absolute values of total electrical conductivity in air shows the highest conductivities for x ≤ 0.5 with a maximum attained in samples with 20 mol.%. ZrO_2_ (Figure 4b), which is consistent with the literature data for same Ce/Zr ratios and temperatures [32,35]. Meanwhile, at 600 °C, the conductivity of CeO_2−δ_ film is significantly higher than that of bulk CeO_2_ reported in [35], but it is similar with electrical conductivity of nanostructured CeO_2−δ_ thin film [56]. At 900 °C, the measured conductivity and literature data for CeO_2_ are equal. Likewise, the conductivities at low *p*O_2_ (~10^−14^ bar) for pure CeO_2_ and 80%-CeO_2_ samples match those that are reported in [32,56]. It is worth noting, for films with 0.2 ≤ *x* ≤ 0.5, the conductivity appears to linearly depend on the ZrO_2_ content in the temperature range of 600–900 °C in air.

Anticipating an Arrhenius-like behavior for the electrical conductivity

*σ* = *σ*_0_ exp(−*E*_a_/*kT*), (11)

the log(*σ*) vs. *T*^−1^ dependences were approximated by a linear fit in different temperature ranges at heating and cooling stages. Table 2 and Figure 4c,d present the derived values for apparent activation energies *E*_a_ and pre-exponential terms *σ*_0_. The values of *E*_a_ and *σ*_0_ are very similar for all mixed Ce_1−x_Zr_x_O_2−δ_ at temperatures increasing from 700 to 900 °C, and they are consistent with literature data for bulk Ce_1−x_Zr_x_O_2_ [35] in the same temperature range.

Taking the lower temperature range (600–700 °C) in consideration, the general trend towards decreasing of *E*_a_ and *σ*_0_ for ceria-rich samples (*x* ≤ 0.5) and, on the contrary, a strong increase of these quantities in CZO-67 and pure ZrO_2_ is notable. The effect appears to become stronger, the greater is the deviation of zirconia fraction from 50 mol.% in both directions. However, it is, in general, weaker for cubic CZOs. This turns to the point, that at the highest temperature range of 800–900 °C, a nearly linear dependence of *E*_a_ on ZrO_2_ content is observed for *x* ≤ 0.67, and the *σ*_0_ dependence is a curve with maximum at 0.33 ≤ *x* ≤ 0.67. The same behavior was also observed under pure Ar flow (*p*O_2_ = 10^−3^ bar) and upon cooling in air from 900 to 600 °C (Table 2). Note that, in Ar, the changes in *E*_a_ for CZO samples with 0.2 ≤ *x* ≤ 0.5 are minor, while the *σ*_0_ parameter significantly increased. The conductivity of CeO_2−δ_ film had undergone the strong decrease of activation energy and *σ*_0_ dropped by one order of magnitude. On the contrary, for CZO-67, the *E*_a_ and *σ*_0_ greatly increased. 

### 3.3. Electrical Conductivity and Non-Stoichiometry of Ce_1−x_Zr_x_O_2−δ_ in Reducing Atmosphere

**Figure 5:**
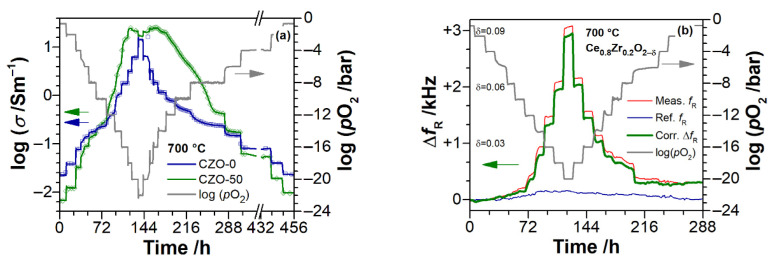
Example for a measurement of the electrical conductivity of CeO_2_ and Ce_0.5_Zr_0.5_O_2_ thin films (**a**) and the change of the resonant frequency Δ*f*_R_ for CZO-20 coated CTGS resonator (**b**) upon stepwise changes of *p*O_2_ at 700 °C. In (**a**) open symbols represent the EIS measurements and solid lines the DC measurements. In (**b**)**,** the raw measurement data (Meas. *f*_R_) are corrected for *f*_R_ of a reference resonator (Ref. *f*_R_), yielding the signal of interest Δ*f*_R_ (Corr. Δ*f*_R_, which is primarily compensated for temperature fluctuations). The frequency shift is given with respect to Meas. *f*_R_ at *p*O_2_ = 0.2 bar. The corresponding non-stoichiometry values, as evaluated by Equation (10), are provided.

Figure 5a,b show examples for typical experimental runs to determine the dependence of the electrical conductivity *σ* and the non-stoichiometry *δ* on the oxygen partial pressure, respectively. An almost instantaneous establishment of the *p*O_2_ in the gas phase upon the stepwise change of log(*p*O_2_) by each −2 was observed for all the isotherms. However, *σ* and *δ* both show a delayed response to the changes of *p*O_2_, as it is seen, e.g., for CZO-50 in Figure 5a or CZO-20 in Figure 5b. Furthermore, differences in the equilibration times and substantial discrepancies (hysteresis) between the equilibrium values of conductivities and non-stoichiometries at same *p*O_2_ in the reduction and oxidation stages are evident. This behavior may be explained by the competition of mechanisms controlling the redox kinetics in CZO: the bulk diffusion of oxygen atoms and surface reaction [41,55]. In quasi-2D samples, like thin films, the rate of surface reaction is usually the limiting factor [41]. However, in practice, the porosity, roughness, and grain boundaries may affect the diffusion of oxygen in the film interior.

The composition of oxygen carrying atmosphere is also important. For example, a 10-times faster relaxation of conductivity was reported for the reduction of CeO_2_ derivatives in Ar-balanced CO-CO_2_ as compared to that in Ar-H_2_-H_2_O mixture [41], and difficulties in regaining oxygen stoichiometry upon oxidation in Ar–H_2_ were reported in [13]. Because of large steps in *p*O_2_ (Δlog(*p*O_2_/bar) = −2, while changes by ca −0.2 are required [41]) and roughness and porosity of the films (increasing with Zr content, but virtually absent in pure CeO_2_ and ZrO_2_), the plausible analysis of observed relaxation of conductivity and non-stoichiometry in the present research is limited.

**Figure 6:**
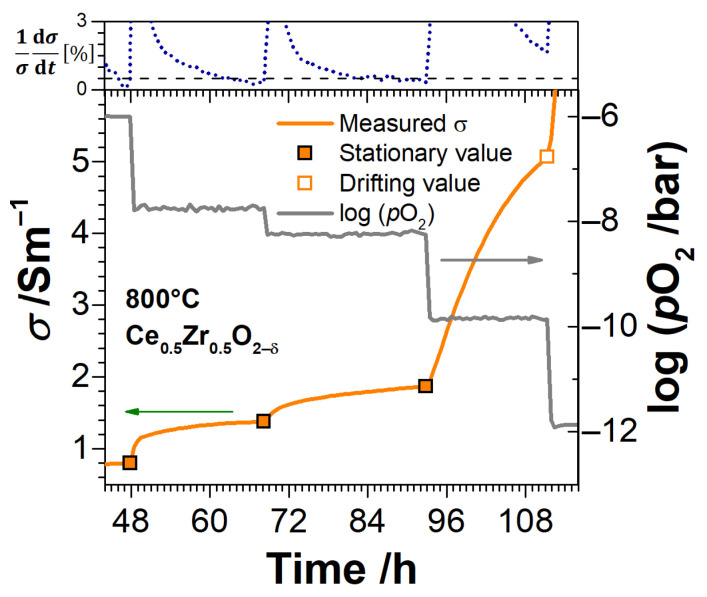
Approach to select stationary and drifting measurement points. In the upper part, the relative change of conductivity with time (blue dotted line) after each log(*p*O_2_) set-point is depicted. If the change is less than 0.5% (black dashed line) of the absolute value *σ* within one hour before the next log(*p*O_2_) set-point, the conductivity is considered stationary. Note the differences in log(*p*O_2_) step heights (less than −2) between *p*O_2_ of 10^−6^ bar and 10^−10^ bar. These originate from the corrections applied due to different operating temperatures of the furnace (800 °C) and *p*O_2_ sensor (700 °C), as explained in Section 2.3.

When considering the time-delayed evolution of conductivity and non-stoichiometry upon changes of *p*O_2_, the experimental data points were divided into “stationary” and “drifting”. The distinction is based on the analysis of time derivatives of these quantities. The measurement point is considered to be stationary if the respective measured quantity has not changed by more than 0.5% in the last hour before the next step in *p*O_2_. In the following, solid symbols show the stationary data points, and open symbols show the drifting ones. It is worth noting, at *p*O_2_ ≥ 10^−6^ bar and *p*O_2_ ≤ 10^−14^ bar, the stationary values of conductivity and non-stoichiometry were usually rapidly attained. The approach to distinction of data points is illustrated with an example for conductivity in Figure 6.

**Figure 7:**
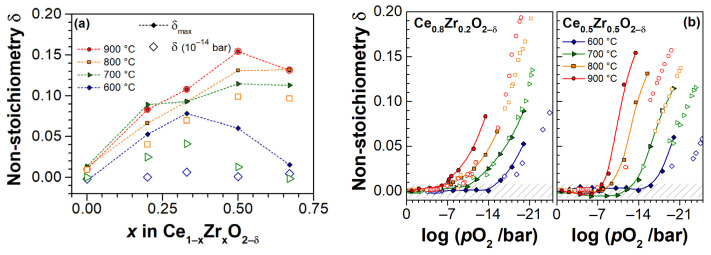
Non-stoichiometry as a function of Zr fraction *x* in Ce_1−x_Zr_x_O_2−δ_: (**a**) maximum non-stoichiometry *δ*_max_ (solid symbols, dashed lines), and *δ* at *p*O_2_ = 10^−14^ bar (open symbols); (**b**) comparison of non-stoichiometries for thin film CZO (solid symbols and lines) with *δ* of bulk CZO from Kuhn, et al. [9] (open symbols). The gray-dashed area depicts the uncertainty range for *δ* of thin films around *δ* = 0.

Figure 7a shows how the oxygen non-stoichiometry depends on the zirconia content *x*. It compares the maximum attained δ at minimum adjusted *p*O_2_ for each isotherm, as well as the non-stoichiometries at *p*O_2_ ≈ 10^−14^ bar for each isotherm (the lowest *p*O_2_ measured at 900 °C, resulting in the largest non-stoichiometry). An increase of reducibility with respect to CeO_2-δ_ is observed for CZO with increasing Zr content, and the highest *δ* are reached in samples with highest ZrO_2_ fractions (CZO-50 and CZO-67). In Figure 7b, our data are compared with the bulk CZO data reported in [9]. The evaluated values of non-stoichiometry for Ce_0.8_Zr_0.2_O_2−δ_ films are quite consistent with the results of [9], although some discrepancies are evident at 600 and 900 °C. For CZO-50 the discrepancies occur at all isotherms. This appears to be another distinct feature of CZO, specifically related to thin film morphology: the character of the dependences is similar, but the threshold *p*O_2_ for the onset of non-stoichiometry in thin films, apparently, is shifted towards higher oxygen partial pressures. Although many factors may contribute to the observed differences (e.g., the measurement methods are different, too low mass of the studied material in nanobalance approach, temperature-related corrections of *p*O_2_ may introduce errors), we note that the shift of threshold *p*O_2_ is rather systematic. This may evidence in a size effect, peculiar for the 2-D nature of thin films, which is plausible in view of the anticipated predominantly surface-controlled redox kinetics in thin films, in contrast to mainly diffusion-controlled processes in bulk materials [41].

**Figure 8:**
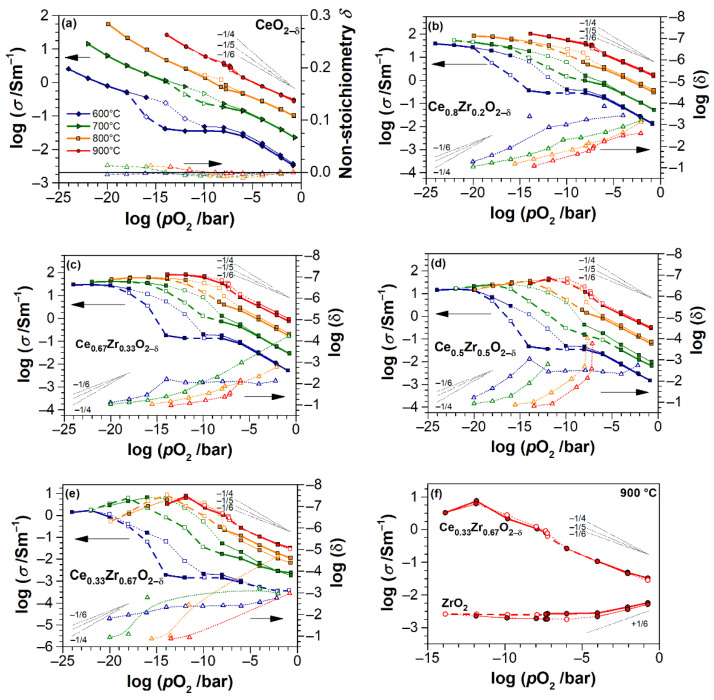
The *p*O_2_-dependences of conductivity *σ* and oxygen non-stoichiometry *δ* of CZO thin films at different isotherms (see legend in (**a**)). For CeO_2−δ_ the oxygen deficiency *δ* is plotted linearly.

**Figure 9:**
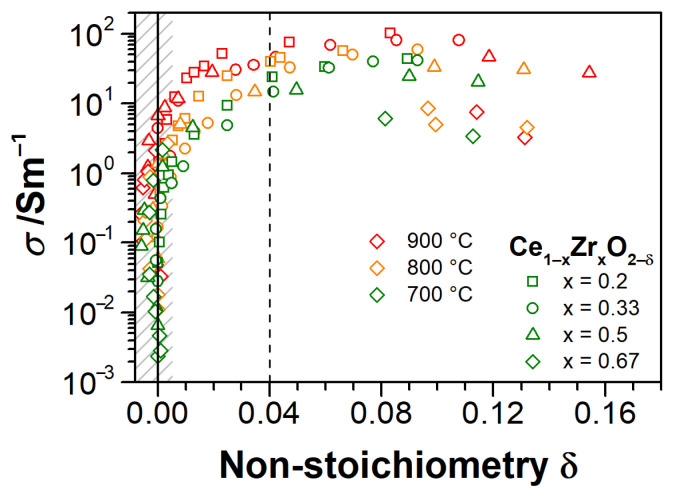
Dependence of the electrical conductivity of Ce_1−x_Zr_x_O_2−δ_ on the oxygen non-stoichiometry δ. The gray-dashed area in the vicinity of δ = 0 indicates the uncertainty range, in which the non-stoichiometry barely changes with decreasing *p*O_2_. The line at δ ≈ 0.04 shows the approximate limit, above which the electrical conductivity for all CZO tends to saturate.

The *p*O_2_-dependences for electrical conductivity of Ce_1−x_Zr_x_O_2−δ_ thin films at each isotherm are shown together with non-stoichiometry data in Figure 8 in order to observe the correlation between these two quantities. The dotted thin lines with slopes of −1/4, −1/5 and −1/6 serve as guides for eyes to follow the (σ, δ) vs. (*p*O_2_)^1/*n*^-relations. The bold and thin curves for conductivity indicate the directions of reduction and oxidation stages, respectively. The dashed lines connect the “drifting” points. Note, that the non-stoichiometry curves in Figure 8 only pertain to the reduction stage for clarity of figures. As said earlier, the hysteresis in *σ* (and *δ*, not shown) can be associated with differences in redox rates at reducing and oxidation of CZO, additionally affected by the Ar–H_2_ atmosphere. The hysteresis decreases with increasing temperature, as seen in Figure 8. In Figure 9, the conductivities are correlated to non-stoichiometry in a condensed manner of log(*σ*) vs. *δ* for 700, 800, and 900 °C isotherms. The data for CeO_2-δ_ films are not included due to scattering of δ values, remaining in the uncertainty range.

We highlight the general behavior of conductivity and non-stoichiometry, as obtained from Figure 4, Figure 7, Figure 8, Figure 9. Consistent with literature data [32,35,55,56,57,63], the conductivity of all the CZO samples increases with temperature. The absolute values of conductivity are composition-dependent, with a maximum for cubic Ce_0.8_Zr_0.2_O_2−δ_ in air (Figure 4b). The conductivities of CZO increase by up to five orders of magnitude (Figure 8) as the *p*O_2_ decreases from 0.2 to 10^−20^ bar at 600 °C and 0.2 to 10^−12^ bar at 900 °C. At least in the temperature range of 700–900 °C, this increase proceeds very rapidly with respect to *δ* in all Ce_1−x_Zr_x_O_2−δ_ films until δ ≈ 0.04 (Figure 9), after which the conductivity becomes less sensitive to an oxygen deficit. This is consistent with observations at lowest *p*O_2_, where the conductivity of CZO tends to a maximum value, the transit through which with respect to temperature and *p*O_2_ is dependent on *x*. This maximum shifts towards lower *p*O_2_ with increasing temperature (Figure 8d,e). The onset of non-stoichiometry also shifts towards lower *p*O_2_ with increasing *T*. In general, the trends in *p*O_2_-dependences of conductivity are equivalently repeated by the trends in non-stoichiometry. At very low *p*O_2_, the non-stoichiometry saturates in concert with the electrical conductivity. The maximum non-stoichiometry of δ ≈ 0.16 was found for the sample with x = 0.5 at *p*O_2_ ≈ 10^−14^ bar and *T* = 900 °C (Figure 7b and Figure 9), whereas the maximum conductivity of about 100 S/m in these conditions was found for *x* = 0.2 (Figure 4b). At *T* = 700–900 °C and in the entire range of *p*O_2_, all of the CZO samples show negative slopes in log(σ)-log(*p*O_2_) and log(δ)-log(*p*O_2_) representations, indicating in predominance of n-type electronic conductivity. However, at 600 °C, the *p*O_2_-independent region of conductivity is observed, which, together with the increased exponents in (*p*O_2_)-dependences for 0.2 ≤ *x* ≤ 0.67, points to conductivity dominated either by impurities or by defect interactions beyond the isolated defect model.

## 4. Discussion

The reducibility of ceria and its Zr-substituted derivatives is primarily dependent on temperature and the zirconia content, as follows from the introduction, defect chemistry, and from data provided in Section 1 and Section 2.1, and Section 3.3, respectively. Furthermore, the defect interactions in CZO can fairly be described in the framework of CeO_2−δ_ and ZrO_2_ defect models. Hence, it is obvious to begin the discussion with samples of edge compositions and compare the observed phenomena in CZO with reference to temperature and oxygen partial pressure.

### 4.1. Electrical Conductivity of ZrO_2_ Thin Films

It should be noted that ZrO_2_ was not in major focus of this research, and the non-stoichiometry data are not measurable with the present method for these thin films. Only the electrical conductivity could be determined together with CZO, but significant scattering of data points precluded the discussion of ZrO_2_ at temperatures *T* ≤ 800 °C.

At 900 °C and 10^−4^–10^−14^ bar, nominally pure zirconia thin films show a characteristic for ionic conductor *p*O_2_-independent conductivity (Figure 8f). A conductivity with a positive slope close to +1/6 is observed at *p*O_2_ > 10^−4^ bar, which suggests the domination of defect electron (hole) transport. The activation energies *E*_a_ at heating from 700 to 800 °C and cooling from 900 to 750 °C in air (1.52 and 1.82 eV, respectively, see Table 2) are consistent with literature data for p-type conducting monoclinic ZrO_2_. For example, *E*_a_ of 1.62 eV is determined for p-type conductivity of ceramic zirconia [64], whereas *E*_a_ in the range of ca. 0.9–1 eV is expected for anion vacancies transport, and is much higher than 2 eV—for n-type electronic conductivity [57,64]. The absolute conductivity value at 900 °C in air is also consistent with that reported for ZrO_2_ ceramics (~10^−2^ S/m; Figure 4b) [64,94].

The ionic conductivity of ZrO_2_ thin films at 900 °C and *p*O_2_ of 10^−4^–10^−14^ bar can be attributed to dominant charge transport via oxygen vacancies, which formed according to [57,62,63]:

(12) $${\mathrm{ZrO}}_{2}{\;=\;\mathrm{Zr}}_{\mathrm{Zr}}^{\text{×}}{\;+\;2V}_{O}^{\;\cdot \cdot }{\;+\;2O}_{i}^{\prime \prime },$$

whereby $V_{O}^{\;\cdot \cdot }$ are more mobile than oxygen interstitials. The p-type conductivity at higher *p*O_2_ can be attributed to the holes that formed by oxygen interstitials at excess oxygen conditions [56]:

(13) $$\frac{1}{2}O_{2}\left(g\right)↔{\;[O}_{i}^{\prime \prime }]\;+\;\left[h^{\cdot }\right].$$

In this case, the concentrations are ${[O}_{i}^{\prime \prime }]\;\approx \;\frac{1}{2}\left[h^{\cdot }\right]\;\gg \;\left[V_{O}^{\;\cdot \cdot }\right]$ or ${[O}_{i}^{\prime \prime }]\;\approx \;\frac{1}{2}\left[h^{\cdot }\right]\;\gg \;\frac{1}{2}\left[e^{\prime \;}\right]$. As *p*O_2_ decreases, the oxidation is weaker, $\left[V_{O}^{\;\cdot \cdot }\right]$ increases, and the ionic conductivity dominates at intermediate *p*O_2_.

### 4.2. Electrical Conductivity and Non-Stoichiometry of CeO_2−δ_ Thin Films

Between 700 °C and 900 °C, the conductivity of CeO_2−δ_ thin films increases with a slope of *p*O_2_-dependence close to −1/6, down to 10^−12^ bar at 900 °C, ca. 10^−14^ bar at 800 °C and 10^−16^ bar at 700 °C (Figure 8a). This implies an n-type electronic conductivity that is consistent with an anticipated small polaron hopping mechanism between the Ce^3+^ and Ce^4+^ sites. That is the conductivity is controlled by the intrinsic compensating defect, i.e., the formation of oxygen vacancies by a reduction of cerium ([${\mathrm{Ce}}_{\mathrm{Ce}}^{\prime }$] = 2[$V_{O}^{\;\cdot \cdot }$] regime), according to Equation (2). In the concerned *p*O_2_ range, *δ* barely changes with *p*O_2_ and it remains within the uncertainty range of the measurement. The higher the temperature, with further decrease of *p*O_2_, the onset of oxygen non-stoichiometry with δ > 0.01 occurs and the electrical conductivity of CeO_2−δ_ appears to take on a steeper *p*O_2_-dependence with negative slopes of which the magnitude is greater than 1/6. This can be understood as an onset of transition to complex defect interactions. Subsequently, the isolated point defect model no longer applies. It can be assumed that, at very low *p*O_2_ (much lower than in this study), the conductivity of CeO_2−δ_ films transits to a regime that is controlled by the formation of either singly ionized oxygen vacancies (Equation (3)) with concentration [${\mathrm{Ce}}_{\mathrm{Ce}}^{\prime }$] = [$V_{O}^{\;\cdot }$] or dimer ${{(\mathrm{Ce}}_{\mathrm{Ce}}^{\prime }V_{O}^{\;\cdot \cdot })}^{\cdot }$ defect associates (Equation (4)). In both cases, the conductivity will vary as (*p*O_2_)^−1/4^. Indeed, as seen in Figure 8a, the slopes of electrical conductivity change between −1/5 and −1/4 at 700–800 °C.

### 4.3. Electrical Conductivity and Non-Stoichiometry of Ce_1−x_Zr_x_O_2−δ_ Thin Films

The effect of Zr addition to ceria is clearly evident from the qualitatively different behavior of electrical conductivity and non-stoichiometry of CZO as compared to that of pure CeO_2−δ_ or ZrO_2_ (Table 2, Figure 4, Figure 7, Figure 8, Figure 9). While, for the latter, a significant contribution of ionic conductivity is inherent with relatively low absolute values of *σ* and pre-exponential factors *σ*_0_ in the whole temperature and *p*O_2_ range, in Ce_1−x_Zr_x_O_2−δ_ thin films, the strongly electronic conductivity prevails at 700–900 °C (Figure 8b–e), along with rapidly attained high degrees of reduction at sufficiently higher oxygen partial pressures. The observed peculiarities imply the superior catalytic properties of CZO thin films, achievable yet at moderate reducing conditions and relatively low temperatures, which are of particular importance for application in automotive TWC and oxygen sensors. However, the improved reducibility cannot be solely assigned to the substitution of Zr for Ce, as further discussed.

The slopes of *p*O_2_-dependences of conductivity for CZO decrease with temperature at high *p*O_2_ from −1/5 at 600 °C to about −1/6 at 900 °C for CZO-20, and from −1/4 to −1/5 for CZO-33, CZO-50, and CZO-67, respectively. This behavior is typical for doped CZO and it reflects the transition from an impurity-controlled (${[A}_{\mathrm{Ce}}^{\prime }$] = 2[$V_{O}^{\;\cdot \cdot }$]) to a reduction-controlled ([${\mathrm{Ce}}_{\mathrm{Ce}}^{\prime }$] = 2[$V_{O}^{\;\cdot \cdot }$]) regime [32,35]. The higher slopes for CZO at low temperatures and high *p*O_2_, when compared to −1/6 for CeO_2−δ_, can, therefore, be explained by the increasing net concentration of impurities (in particular, Al_2_O_3_ and CaO) with increasing Zr fraction in the former. Despite the scattered data, one can observe that the oxygen non-stoichiometry of CZO also closely follows the −1/5 and −1/4 dependences in these conditions at temperatures of 700–900 °C (e.g., in Figure 8b,c). However, the non-stoichiometries of all samples are too low for a reliable correlation with conductivity in this *p*O_2_ range.

Notably, for all Ce_1−x_Zr_x_O_2−δ_ samples, the threshold *p*O_2_ for the onset of oxygen non-stoichiometry is five to six orders of magnitude higher, as compared to pure CeO_2−δ_ (e.g., 10^−10^–10^−11^ bar for CZO-20, CZO-33, and CZO-50 vs. 10^−15^ bar for CZO-0, as seen from Figure 8b–d and a, respectively, at 700 °C). Furthermore, this threshold oxygen partial pressure shifts towards higher *p*O_2_ with an increasing temperature. Prior to threshold pressure, at an intermediate *p*O_2_ of ca. 10^−6^–10^−10^ bar, a conductivity dependence that is close to (*p*O_2_)^−1/6^ is observed for Ce_1−x_Zr_x_O_2−δ_ at 700 °C, although changes in δ are still less than 0.01. Below the threshold *p*O_2_, the oxygen non-stoichiometry rapidly increases with decreasing *p*O_2_, and the conductivity shows the transition to a steeper slope closer to −1/4 (depending on isotherm, the *p*O_2_ ranges from 10^−11^–10^−16^ bar (700 °C) to 10^−7^–10^−11^ bar (900 °C) are considered). The transitions are evident for CZO-50 and CZO-67. Furthermore, the log(*δ*)–log(*p*O_2_) dependences at 800–900 °C in Figure 8d,e also show a slope close to −1/4 for CZO-67 and CZO-50. Upon further reduction, the conductivity tends to saturate in concert with non-stoichiometry and it reaches a maximum (CZO-50 and CZO-67). Surpassing the maximum, at the lowest *p*O_2_, the conductivity dependence on oxygen partial pressure of CZO-50 and CZO-67 turns to a positive slope of about +1/6. Combining these observations and assuming a conventional defect model for bulk CZO (Section 2.1), the observed behavior of electrical conductivity and non-stoichiometry of CZO with increasing temperature (700–900 °C) and decreasing *p*O_2_ can be summarized, as follows:

- Impurity-mediated conductivity at high *p*O_2_ ≥ 10^−6^ bar and 700 °C, governed by the electroneutrality condition ${[A}_{\mathrm{Ce}}^{\prime }$] = 2[$V_{O}^{\;\cdot \cdot }$], although with increasing temperature, the conductivity turns closer to a (*p*O_2_)^−1/6^-dependence with the electroneutrality condition [${\mathrm{Ce}}_{\mathrm{Ce}}^{\prime }$] = 2[$V_{O}^{\;\cdot \cdot }$].
- At intermediate *p*O_2_ (10^−12^–10^−6^ bar at 700 °C), the regime transits to [${\mathrm{Ce}}_{\mathrm{Ce}}^{\prime }$] = 2[$V_{O}^{\;\cdot \cdot }$], as indicated by the changing slope from −1/4 to −1/6, although the non-stoichiometry is still low (ca. 0.01).
- At further reduced conditions with *p*O_2_ between 10^−18^ and 10^−12^ bar at 700 °C, strong non-stoichiometry starts and the onset of defect interactions leads to (*p*O_2_)^−1/4^-dependence of conductivity. Complex interactions between Ce^3+^ cations and oxygen vacancies are assumed. The conductivity transits to [$V_{O}^{\;\cdot }$] = [${\mathrm{Ce}}_{\mathrm{Ce}}^{\prime }$] controlled regime, which is characterized by the formation of either singly ionized oxygen vacancies (Equation (3)) or ${{(\mathrm{Ce}}_{\mathrm{Ce}}^{\prime }V_{O}^{\;\cdot \cdot })}^{\cdot }\;$dimers (Equation (4)).
- At lowest *p*O_2_ (<10^−18^ bar at 700 °C), the oxygen non-stoichiometry rapidly exceeds 0.04 (Figure 9), and [${\mathrm{Ce}}_{\mathrm{Ce}}^{\prime }$] ≥ [${\mathrm{Ce}}_{\mathrm{Ce}}^{\text{×}}$] holds. The saturation of *δ* and conductivity occurs, inferring complex defect interactions that eventually lead to transit through a maximum of conductivity for CZO with the largest Zr content and a positive slope of ca. +1/6 (p-type conductivity [32]). The latter can be attributed either to strong associations between univalent Ce interstitials and reduced Ce^3+^ or to a trapping of excess electrons by oxygen vacancies [57] (i.e., essentially the formation of dimer and trimer defect associates), both suppressing the electronic contribution to the conductivity.

The described impetuous rise of electrical conductivity and defect interactions, occurring in CZO already at rather high *p*O_2_, in CeO_2−δ_ thin films only onset at very low *p*O_2_. This is consistent with an anticipation of temperature- and Zr-dependent increase of intrinsic contribution in the conductivity by the facilitated Ce^4+^/Ce^3+^ reduction in Zr-substituted ceria. When combining the indications of Figure 8 and Figure 9, one can infer that the electrical conductivity of Ce_1−x_Zr_x_O_2−δ_ dramatically increases by about five orders of magnitude already at low non-stoichiometries (δ ≤ 0.02), following which at δ ≥ 0.04 the electrical conductivity (in terms of orders of magnitude) barely depends not only on non-stoichiometry, but, probably, also on temperature and zirconia content.

At 600 °C and at intermediate oxygen partial pressures of 10^−8^–10^−14^ bar, an ionic-like *p*O_2_-independent electrical conductivity occurs in CeO_2-*δ*_ and CZO films (Figure 8). It can be, primarily, associated with the impurity-mediated electrical conductivity, resulting in ionic contribution via the oxygen vacancies formed according to Equations (6) and (7), although the electronic contribution at oxidizing conditions (*p*O_2_ ≥ 10^−6^ bar) is puzzling. We note that in starting CeO_2_ and ZrO_2_ alumina (Al_2_O_3_) was the major impurity. A similar unusual course of low-temperature conductivity with a plateau at intermediate *p*O_2_ was reported for Y_2_O_3_-doped Ce_1−x_Zr_x_O_2−δ_ in [34]. It was assumed to result from complicated interactions of reduced Ce^3+^ with acceptor ions and/or oxygen vacancies, although no exact relations were suggested. Presumably, also the reductive nature of Fe_2_O_3_ impurity could contribute to this unusual behavior. Furthermore, the size and disorder effects in thin films may enhance the ionic conductivity [52,55]. Hence, accounting for the morphology of thin films studied herein, the influence of porosity and roughness (increasing with Zr fraction) should not be excluded. The effective charge of subsurface atoms in thin films with large surface area-to-volume ratios may be different from that in bulk materials [54,55,56,95], e.g., the surface oxygen vacancies may have fractional charges, which leads to conductivities with slopes between −1/3 and −1/2 at reducing conditions. Such steeper slopes are actually observed for Zr-rich CZO-33, CZO-50 and CZO-67 samples at 600 °C upon transition from conductivity plateau at *p*O_2_ = 10^−14^ bar (Figure 8c–e).

We must note that, in the present research, no studies that were specifically relevant to morphological effects on the conductivity of CZO thin films were carried out. The proposed defect interaction model is built with the approximation of bulk defect chemistry of reduced CeO_2_. The appropriate accounting for the morphological peculiarities, in particular, enhanced surface area-to-volume ratio of rough thin films, needs a further extension of the defect model. This would become possible if the appropriate comparisons of the electrical properties of thin film Ce_1−x_Zr_x_O_2−δ_ with those of bulk materials used for their production were performed. This will be the topic for our forthcoming research.

## 5. Conclusions

A linking of redox and electronic behavior of thin film Ce_1−x_Zr_x_O_2−δ_ solid solutions as prospective catalyst materials for use as an oxygen storage component in automotive three-way catalyzers is discussed. A unique nano-thermogravimetric method that is based on the resonant nanobalance approach for high-temperature characterization of oxygen non-stoichiometry of ceria-zirconia solid solutions in the broad range of oxygen deficiencies was implemented. The method enabled the measurements of non-stoichiometries δ, even below 0.01 for thin films having material masses as small as some tens of micrograms, which are inaccessible by conventional thermogravimetric methods.

The electrical and redox properties of Ce_1−x_Zr_x_O_2−δ_ solid solutions are substantially different from those of thin films of the edge compositions, i.e., CeO_2−δ_ and ZrO_2_, as the former showed strongly n-type electronic conductivity. The highest conductivity was obtained in ceria-rich thin films (*x* = 0.2; 0.33), whereas the largest oxygen non-stoichiometries were observed for Zr-rich films (*x* = 0.5; 0.67). The reducibility of Ce_1−x_Zr_x_O_2−δ_ was greatly enhanced when compared with pure CeO_2-δ_. The threshold *p*O_2_ for the onset of the non-stoichiometry is up to seven orders of magnitude higher than that of pure CeO_2-δ_, and it shifts towards higher *p*O_2_ with increasing temperature. The maximum non-stoichiometry δ ≈ 0.16 was measured for Ce_0.5_Zr_0.5_O_2−δ_ at 900 °C and *p*O_2_ ≈ 10^−14^ bar, which is fairly consistent with the literature. Overall, the conductivities of the studied CZO thin films have changed by about four orders of magnitude with respect to *p*O_2_ changes within 0.2 bar to 10^−24^ bar. When combined with the temperature-related conductivity increase, the increases by five orders of magnitude were determined. In the case of pure CeO_2−δ_, the total changes were less than four orders of magnitude. A tendency towards a composition-dependent saturation of non-stoichiometry and electrical conductivity was observed for Ce_1−x_Zr_x_O_2−δ_. This is consistent with the theoretically anticipated behavior of polaronic transport in ceria derivatives, when described in the framework of the isolated defect model. However, although, overall, a correlation between the temperature- and *p*O_2_-dependences of oxygen non-stoichiometry and electrical conductivity of CZO was evident, the comprehensive analysis of these relations infers that complex defect interactions in the Ce_1−x_Zr_x_O_2−δ_ must be taken into account. Apart from impurity effects, these include the possible dimer and trimer associates between reduced Ce cations and oxygen vacancies. Furthermore, size and disorder effects, as they are characteristic for thin films, on the redox behavior of Ce_1−x_Zr_x_O_2−δ_ are suspected.

## Figures and Tables

**Table 1:** Characteristics of Ce_1−x_Zr_x_O_2−δ_ thin film samples.

Nominal ZrO_2_ Content in mol.%	Actual Composition (EDS)	Electrical Conductivity		Nanobalance Approach		
		*t*/µm	*L/*mm	*d*_CTGS_/µm	*d*_CZO_/µm	*m*_0_/µg
**0**	CeO_1.91_ *	1.98	3.9	274.5	3.72	461.4
20	Ce_0.80_Zr_0.20_O_1.94_	1.97	3.9	246.5	6.09	834.5
33	Ce_0.67_Zr_0.33_O_1.91_	2.13	3.2	283.4	2.78	369.6
50	Ce_0.51_Zr_0.49_O_1.90_	5.06	3.9	255.0	3.61	461.4
67	Ce_0.35_Zr_0.65_O_2.04_	3.94	1.9	257.6	4.02	491.4
100	n/a	2.67	1.9	n/a	n/a	n/a
Ref. CTGS	n/a	n/a	n/a	294.5	0	0

*t*, *d*_CZO_—thicknesses of CZO films; *L*—distance between electrodes; *d*_CTGS_—thickness of CTGS plates; *m*_0_—“loaded” mass of CZO layer. * Inclusions of Ce_2_O_3_ detected.

**Table 2:** Apparent activation energies *E*_a_ (in eV) and pre-exponential factors *σ*_0_ (in MS/m) of the electrical conductivity of Ce_1−x_Zr_x_O_2_ thin films in air and in argon.

Sample	600–700 °C (Air)		600–700 °C (Argon)		700–800 °C (Air)		800–900 °C (Air)		900–750 °C (Air, Cool.)		750–600 °C (Air, Cool.)	
	*E*_a_	*σ*_0_	*E*_a_	*σ*_0_	*E*_a_	*σ*_0_	*E*_a_	*σ*_0_	*E*_a_	*σ*_0_	*E*_a_	*σ*_0_
CZO-0	1.56	4.2	1.33	0.53	1.32	0.191	1.28	0.125	1.32	0.151	1.42	0.5
CZO-20	1.62	19	1.56	25.7	1.56	7.35	1.44	2.64	1.43	3.01	1.59	19
CZO-33	1.57	6.42	1.60	22.8	1.62	8.62	1.55	5.12	1.57	6.2	1.63	12.6
CZO-50	1.63	2.96	1.69	21.6	1.68	6.18	1.62	3.74	1.63	4.18	1.69	7.94
CZO-67	1.06 *	0.0004 *	1.68	2.36	1.66	0.88	1.76	1.46	1.64	0.381	1.43	0.0368
CZO-100	-	-	-	-	1.52	0.022	2.32	70.6	1.82	0.441	-	-

* The value is not certain due to lack of data points.

## Data Availability

All relevant data presented in the article are stored according to institutional requirements and as such are not available online. However, all data used in this manuscript can be made available upon request to the authors.
